# The Making of the Modern Practitioner

**Published:** 1912-02-17

**Authors:** 


### THE MAKING OF THE MODERN PRACTITIONER.

### Degrees and Diplomas.—II.

There are large numbers of people who gauge the scientific attainments of the doctors in their locality by the number of initials which they can or do append to their names. For these a string of eight letters (namely, M.R.C.S., L.R.C.P.) is a sign of knowledge four times as profound as that implied by two letters (namely, M.D.). Those who are interested in hospital work as a rule do not make such curious mistakes as this; but even trained nurses and others in daily contact with doctors have the vaguest ideas about the mysterious letters after the names of medical men.

### An Old Problem.

What practitioners should be addressed as "Dr." and what as "Mr."? First, let it be stated that by a convention which obtains in this country he who practises as a surgeon (in any branch of surgery) is addressed as "Mr.," even though he may actually be a Doctor of Medicine (M.D.). As a rule surgeons are not M.D., for those who intend to take up surgery as a specialty carefully refrain from acquiring that degree. They often become M.B.; but as a rule, to which there are exceptions, they go no further in university status.

This brings us to the relation of the M.B. and the M.D. The latter is merely a development of the former. The M.B. degree (Bachelor of Medicine) of any British university is a fully registrable qualification, and many practitioners (surgeons and others as well) never proceed to the higher degree. To obtain the latter is at some universities a matter of examination; at others a matter of original research. Taking a broad view, it must be allowed that there is no real superiority implied in being M.D. over him who is M.B. of the same university. There are wide differences in the standards required at the individual universities; but the idea that the M.D. is *ipso facto* a cleverer or more experienced man than the M.B. is quite a delusion.

Now he who is M.D. is properly addressed as Dr.; that is beyond dispute. As a convention, once more, he who is M.B. is granted the courtesy title of Dr., to which he has strictly no right. Should such a M.B. be a surgeon, he does not take advantage of this courtesy; but otherwise as a rule he does. To discuss *in extenso* the merits of these degrees as granted at rival universities would be to venture on very thin ice. All that can safely be said is that there are wide variations in the standard of proficiency required for the degrees of the different universities; that in the estimation of laymen the M.D. of London University is the highest of these honours, and that the authorities and graduates of Oxford, Cambridge, and Edinburgh Universities are not disposed to concede this fancied superiority.

### An Export Degree.

There is one foreign doctorate which is possessed by a good many British practitioners. This is the M.D. (Bruxelles), a distinction which is manufactured entirely for export, since it does not qualify its possessor to practise his profession in Belgium itself. Those who have acquired this degree, however, uphold it as a sign of a searching test of knowledge; and, after all, they should know more about it than stay-at-homes, who have not submitted themselves to foreign examiners.

### British Diplomas.

So much for degrees. If a practitioner decides not to seek a degree, or, having sought one, fails to achieve it for any reason, he can still claim admission to the register by obtaining certain licences and diplomas. The best known are those granted jointly by the English Colleges of Physicians and Surgeons, and the abbreviation of these is M.R.C.S., L.R.C.P. This is the qualification for registration which is possessed by the majority of practitioners in England; in Scotland and Ireland there are diplomas granted by similar Colleges.

The possession and registration of these diplomas does not confer the status of "Dr.," nor is there any convention in the medical profession to that effect. The public, however, has got into the habit of addressing every practitioner as "Dr.," and some diploma-holders adopt the prefix as the most convenient way of swimming with the tide. As a rule, in strictly professional intercourse such a diplomee does not, and decidedly should not, use the style of "Dr.," even though for convenience he may do when dealing with the public.

There are also certain higher diplomas, only to be obtained by passing much more stringent examination tests. Of these the most esteemed are the Fellowship of the College of Surgeons (F.R.C.S.) and the Fellowship and Membership of the College of Physicians (F.R.C.P., M.R.C.P.). The English are the most esteemed of these diplomas, though the Scotch and Irish are perhaps more useful in the respective lands of their acquisition. These higher diplomas do not, any more than the ordinary diplomas, confer any status as "Dr." The F.R.C.S. may be fairly described as the premier surgical qualification of the day; in this respect it outranks the most respected of doctorates. In London, and in most very large towns, it is now next to impossible to obtain an appointment as visiting surgeon to a reputable hospital unless one is F.R.C.S. This does not hold good to a like extent in medicine, though many hospitals require that their physicians shall be M.R.C.P. (at least), as well as M.D. or M.B.

In addition to the foregoing there are other registrable qualifications. Thus the Licentiate of the Society of Apothecaries (L.S.A.) is a full qualification; not very many medical men now take it, since these licentiates are undoubtedly handicapped thereby in competition for appointments. Then there is the F.F.P.S.G.—a distinction signifying the Fellowship of the Faculty of Physicians and Surgeons of Glasgow. There are also certain rarer degrees, such as M.C. (Master in Surgery).

